# Precision surgical oncology for hepatocellular carcinoma

**DOI:** 10.3389/fonc.2026.1957598

**Published:** 2026-09-28

**Authors:** Takehiro Okabayashi, Motoyasu Tabuchi, Teppei Tokumaru, Sunao Uemura, Shuta Tamura

**Affiliations:** Department of Gastroenterological Surgery, Kochi Health Sciences Center, Kochi, Japan

**Keywords:** artificial intelligence, digital twin, hepatocellular carcinoma, liquid biopsy, precision surgical oncology, radiomics

## Abstract

Hepatocellular carcinoma (HCC) is a heterogeneous tumor–liver–host disease in which treatment selection depends on tumor burden, liver function, portal hypertension, performance status, and patient preferences. Although hepatic resection and other established treatment modalities remain central to curative and disease-controlling strategies, emerging biological and digital technologies may provide additional information beyond conventional clinical assessment. However, their prognostic performance does not necessarily establish clinical utility, and many remain insufficiently validated for routine treatment selection. This narrative review examines the evolving role of precision-oriented approaches in HCC, including molecular and immune profiling, radiomics, circulating tumor DNA, artificial intelligence, and digital pathology. We classify these approaches into three evidence tiers: established clinical practice, investigational clinical tools, and future hypotheses. We then propose Precision Surgical Oncology as an author-developed, hypothesis-generating framework in which guideline-based assessment is followed by biological and host-risk refinement, multidisciplinary interpretation, individualized treatment within established therapeutic pathways, and longitudinal reassessment. Precision Surgical Oncology is intended to complement, rather than replace, evidence-based HCC management. Its future clinical value will depend on prospective validation, reproducibility, defined clinical actionability, equitable implementation, and demonstration of improved patient outcomes.

## 1 Introduction

Hepatocellular carcinoma (HCC) remains a major global health burden and accounted for a substantial proportion of liver cancer mortality in 2022 (1). Its clinical management is complex because tumor biology, underlying liver disease, and patient characteristics vary considerably among individuals. Current guidelines emphasize multidisciplinary, risk-adapted treatment based on tumor burden, liver function, portal hypertension, performance status, and patient preferences, with hepatic resection, liver transplantation, locoregional therapy, and systemic therapy selected according to disease stage and clinical context (2–4). However, conventional treatment algorithms remain predominantly anchored in anatomical and physiological parameters, whereas postoperative recurrence and treatment response are also determined by heterogeneous tumor biology and host resilience (5).

Recent advances in molecular profiling, radiomics, artificial intelligence (AI), digital pathology, and liquid biopsy have created opportunities to characterize tumor aggressiveness and treatment response beyond conventional staging (6–9). Nevertheless, most of these technologies remain investigational, with limitations in assay standardization, external validation, reproducibility, and clinical actionability (7–9). Accordingly, they should currently complement rather than replace established HCC decision pathways.

In this narrative review, we propose Precision Surgical Oncology as an author-proposed framework that integrates established clinical assessment with emerging biological and digital information to support multidisciplinary decision-making. We distinguish validated clinical practice from investigational tools and future hypotheses, and discuss how this framework may evolve toward adaptive, patient-centered surgical oncology.

## 2 Methods

This article was designed as a narrative review and conceptual perspective rather than a systematic review or meta-analysis. A targeted literature search was conducted to identify clinically relevant and emerging evidence concerning precision-oriented management of hepatocellular carcinoma (HCC), with particular emphasis on surgical decision-making and multidisciplinary care.

PubMed/MEDLINE, Embase, and Web of Science were searched for publications available through June 2026 using combinations of the following terms: *hepatocellular carcinoma*, *liver cancer*, *hepatic resection*, *liver transplantation*, *ablation*, *precision oncology*, *precision surgery*, *molecular profiling*, *liquid biopsy*, *circulating tumor DNA*, *radiomics*, *artificial intelligence*, *digital pathology*, *immune profiling*, *minimal residual disease*, and *multidisciplinary decision-making*. Additional relevant studies were identified by screening the reference lists of key articles and recent clinical practice guidelines.

Priority was given to recent clinical practice guidelines, randomized controlled trials, prospective studies, large observational studies, systematic reviews, and high-quality translational studies. Landmark earlier studies were retained when necessary to establish the clinical or conceptual basis of current practice. Evidence was critically interpreted according to its degree of clinical validation and actionability. Established clinical practice was distinguished from investigational clinical tools and future hypotheses, and emerging biomarkers or technologies were not considered substitutes for guideline-based assessment of liver function, portal hypertension, tumor burden, performance status, and transplant eligibility.

Because this review proposes an author-developed Precision Surgical Oncology framework, the conceptual components presented herein should be regarded as a hypothesis-generating framework rather than validated clinical guidance.

## 3 The current foundation of HCC management: guideline-based multidisciplinary decision-making

The evolution of HCC management can be viewed as a transition from anatomy-focused surgery to multidisciplinary care and, more recently, toward precision-informed decision-making (**Figure 1**). This evolution does not replace established treatment pathways; rather, it provides an opportunity to incorporate biological, imaging, and patient-specific information into multidisciplinary clinical judgment.

**Figure 1:**
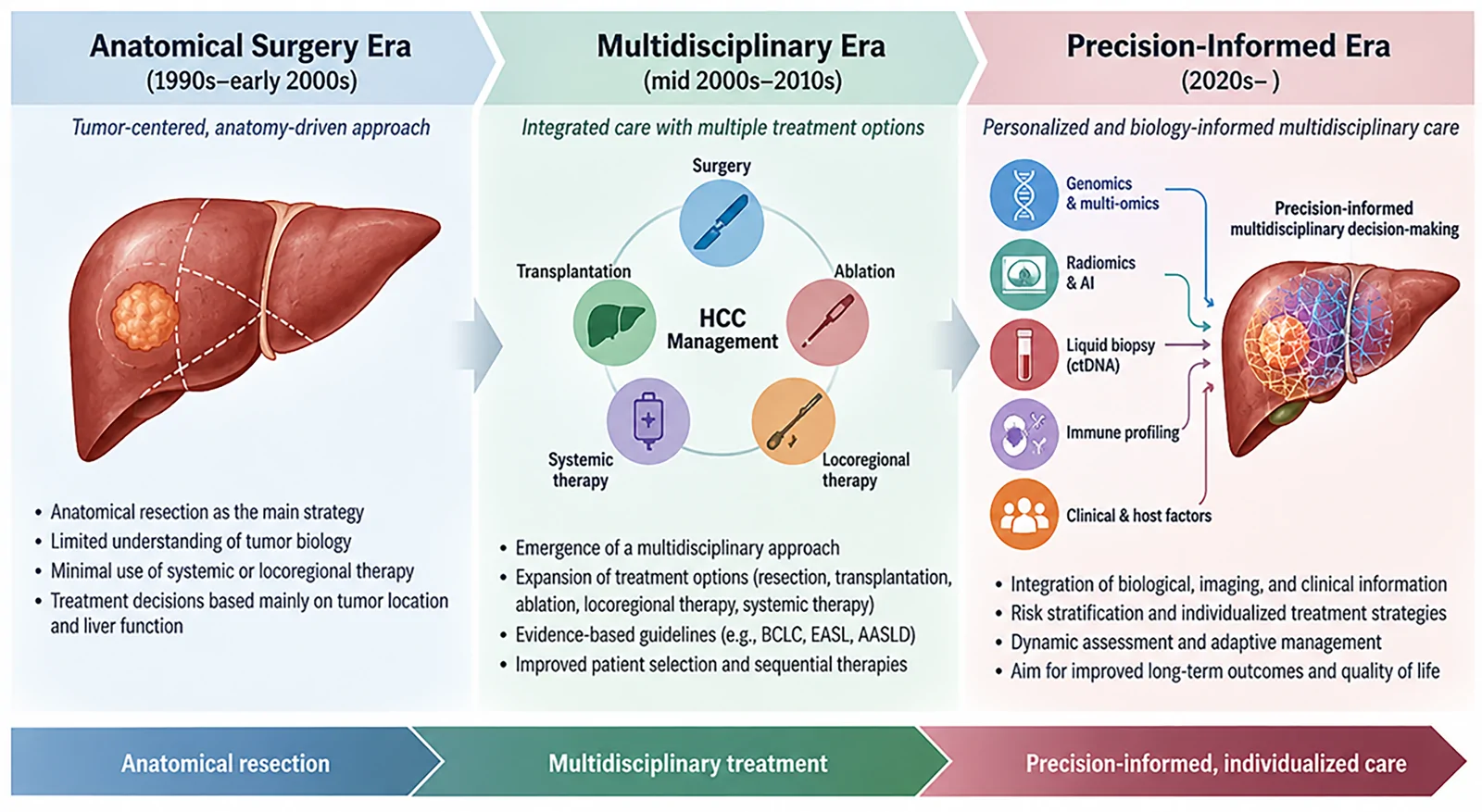
Evolution of HCC management: From anatomical surgery to multidisciplinary and precision-informed care. HCC management has evolved from anatomy-focused surgical treatment toward multidisciplinary care incorporating resection, transplantation, ablation, locoregional therapy, and systemic treatment. The emerging precision-informed approach builds on these established pathways by incorporating biological, imaging, molecular, and patient-specific information to refine risk assessment and multidisciplinary decision-making. Importantly, precision-informed care is intended to complement, rather than replace, guideline-based treatment strategies.

### 3.1 HCC as a tumor–liver–host disease

Hepatocellular carcinoma (HCC) should be regarded not simply as a localized malignancy but as a tumor–liver–host disease, in which treatment decisions are determined by the interaction between tumor characteristics, underlying liver disease, and patient-specific factors. Tumor burden, vascular involvement, liver function, portal hypertension, performance status, and the etiology of chronic liver disease collectively influence prognosis and treatment feasibility (2–4). Patient frailty, nutritional and functional status, and individual preferences are also increasingly recognized as important components of treatment selection (5, 6). Thus, optimal HCC management requires multidimensional assessment rather than tumor anatomy alone.

### 3.2 Established criteria for surgical candidacy

Hepatic resection remains an established potentially curative treatment for appropriately selected patients. Surgical candidacy is primarily determined by tumor resectability, liver functional reserve, portal hypertension, future liver remnant, tumor location, and vascular involvement (2–4). These parameters remain the foundation of surgical decision-making and should not be replaced by emerging biomarkers or computational technologies. Rather, biological information should be considered complementary to this established clinical assessment.

### 3.3 Established curative-intent strategies

For patients with potentially curable HCC, hepatic resection, liver transplantation, and local ablation represent the principal curative-intent strategies, with selection determined by tumor characteristics, liver function, portal hypertension, transplant eligibility, and patient-related factors (2–4). Liver transplantation is particularly important for patients with HCC and clinically significant underlying liver dysfunction, and contemporary transplant practice increasingly incorporates tumor response and biological risk into selection and prioritization (10).

### 3.4 Locoregional and systemic therapy

Patients who are not candidates for immediate curative treatment may undergo locoregional or systemic therapy according to disease stage and clinical characteristics. Transarterial therapies, hepatic arterial infusion chemotherapy, radiotherapy, immune checkpoint inhibitor-based regimens, and molecularly targeted therapies constitute important components of modern HCC management (2–4). Importantly, perioperative and adjuvant systemic therapy remains an evolving field; the updated IMbrave050 analysis did not demonstrate a sustained recurrence-free survival benefit and does not support routine adjuvant atezolizumab plus bevacizumab (9).

### 3.5 Multidisciplinary treatment sequencing

Because HCC encompasses diverse tumor and liver conditions, treatment should be determined through multidisciplinary discussion rather than by a single modality. Surgery, locoregional therapy, systemic therapy, and, in selected circumstances, conversion surgery or repeat treatment may be sequenced according to treatment response, liver function, and evolving clinical status (2–4). This guideline-based multidisciplinary framework constitutes the essential foundation upon which Precision Surgical Oncology should be built. Emerging biological and digital tools should therefore refine, rather than replace, established clinical decision-making.

## 4 Biological heterogeneity and emerging tools for precision stratification

### 4.1 Tumor heterogeneity and molecular biology

Hepatocellular carcinoma (HCC) is characterized by substantial intertumoral and intratumoral heterogeneity, arising from diverse etiologies, chronic liver injury, and ongoing clonal evolution (5). Recurrent genomic alterations involving the TERT promoter, TP53, and CTNNB1, together with distinct molecular and histological subclasses, contribute to differences in tumor behavior, immune phenotype, and therapeutic response (11). Importantly, molecular profiling may provide prognostic or biological information beyond conventional anatomical staging, but its routine use for surgical selection remains unestablished. Thus, molecular characteristics should currently be interpreted as complementary biological information rather than independent determinants of treatment.

### 4.2 Microvascular invasion and recurrence biology

Microvascular invasion (MVI), satellite nodules, poor differentiation, and vascular invasion are strongly associated with early recurrence and adverse outcomes following curative-intent treatment (12). Preoperative models incorporating clinical, laboratory, and imaging characteristics, including radiomics, may improve the prediction of MVI. However, methodological heterogeneity and limited external and prospective validation remain important barriers to clinical implementation (13). Therefore, MVI prediction should currently be regarded as a prognostic and risk-stratification tool rather than an established determinant of wider resection or operative extent.

### 4.3 Host biology

Tumor biology alone does not determine treatment benefit. Frailty, sarcopenia, nutritional status, functional reserve, biological aging, and overall resilience influence postoperative complications, treatment tolerance, and long-term outcomes (14). Patient goals and preferences should therefore be incorporated alongside tumor and liver characteristics, particularly in older or medically complex patients.

### 4.4 Molecular and immune profiling

Genomic, transcriptomic, and immune profiling provides increasingly detailed characterization of the tumor microenvironment (TME). Single-cell and spatial approaches have demonstrated substantial heterogeneity in tumor and immune-cell populations, potentially identifying prognostic and predictive biomarkers (11, 15). Nevertheless, most molecular and immune signatures remain investigational, and their ability to improve treatment outcomes through biomarker-guided surgical decision-making has not been established.

### 4.5 Liquid biopsy and ctDNA

Liquid biopsy, particularly circulating tumor DNA (ctDNA), offers a minimally invasive approach for assessing tumor-derived molecular signals and potential minimal residual disease (MRD). Recent meta-analytic evidence indicates that postoperative ctDNA positivity is associated with increased recurrence risk after curative-intent treatment (16). However, reported sensitivity varies substantially among studies, reflecting differences in assay design, tumor shedding, and analytical methods. Accordingly, ctDNA currently represents a promising investigational biomarker for recurrence-risk stratification and MRD assessment, rather than a validated basis for altering surgery or adjuvant treatment in routine HCC practice.

Collectively, these biological approaches expand the information available for precision stratification, but their clinical role remains primarily prognostic, investigational, or hypothesis-generating. Their integration into treatment decisions requires prospective validation demonstrating that biomarker-guided interventions improve clinically meaningful outcomes.

## 5 Emerging digital technologies: promise, limitations, and clinical readiness

To distinguish established clinical practice from emerging technologies, the components of Precision Surgical Oncology are classified into three evidence tiers (**Table 1**). Guideline-based clinical assessment remains the foundation of HCC treatment, whereas radiomics, ctDNA, molecular and immune profiling, AI, and digital pathology are currently investigational. Multi-omics integration, digital twins, digital biomarkers, and learning health systems represent future hypotheses whose clinical utility remains to be established. Importantly, investigational technologies are intended to complement, rather than replace, guideline-based clinical assessment. Their clinical value should ultimately be determined by prospective validation and demonstrable improvement in patient outcomes.

**Table 1:** Evidence tiers and clinical actionability of precision tools in hepatocellular carcinoma.

Evidence tier	Precision tool	Potential clinical role	Current actionability
Established	Liver reserve, portal hypertension, tumor burden, resectability, performance status, transplant criteria	Treatment selection and surgical candidacy	Direct clinical decision
Investigational	Radiomics	Risk stratification and prediction of recurrence/MVI	MDT discussion/research
Investigational	ctDNA/liquid biopsy	MRD and recurrence-risk assessment	Research/trial enrollment
Investigational	Genomic/immune profiling	Biological characterization and treatment-response assessment	Research/trial enrollment
Investigational	AI/digital pathology	Risk and biological phenotype prediction	Decision support/research
Future hypotheses	Multi-omics integration	Dynamic biological phenotyping	Not currently actionable
Future hypotheses	Digital twins/multimodal AI	Treatment simulation and individualized prediction	Not currently actionable
Future hypotheses	Digital biomarkers/wearables/learning health systems	Continuous monitoring and evidence generation	Not currently actionable

The clinical readiness of precision technologies varies substantially across the HCC treatment continuum (**Figure 2**). Established clinical assessment remains the basis of treatment selection, whereas radiomics, ctDNA, molecular and immune profiling, AI, and digital pathology remain investigational. Multi-omics integration, digital twins, and digital biomarkers represent future hypotheses requiring further validation before clinical adoption.

**Figure 2:**
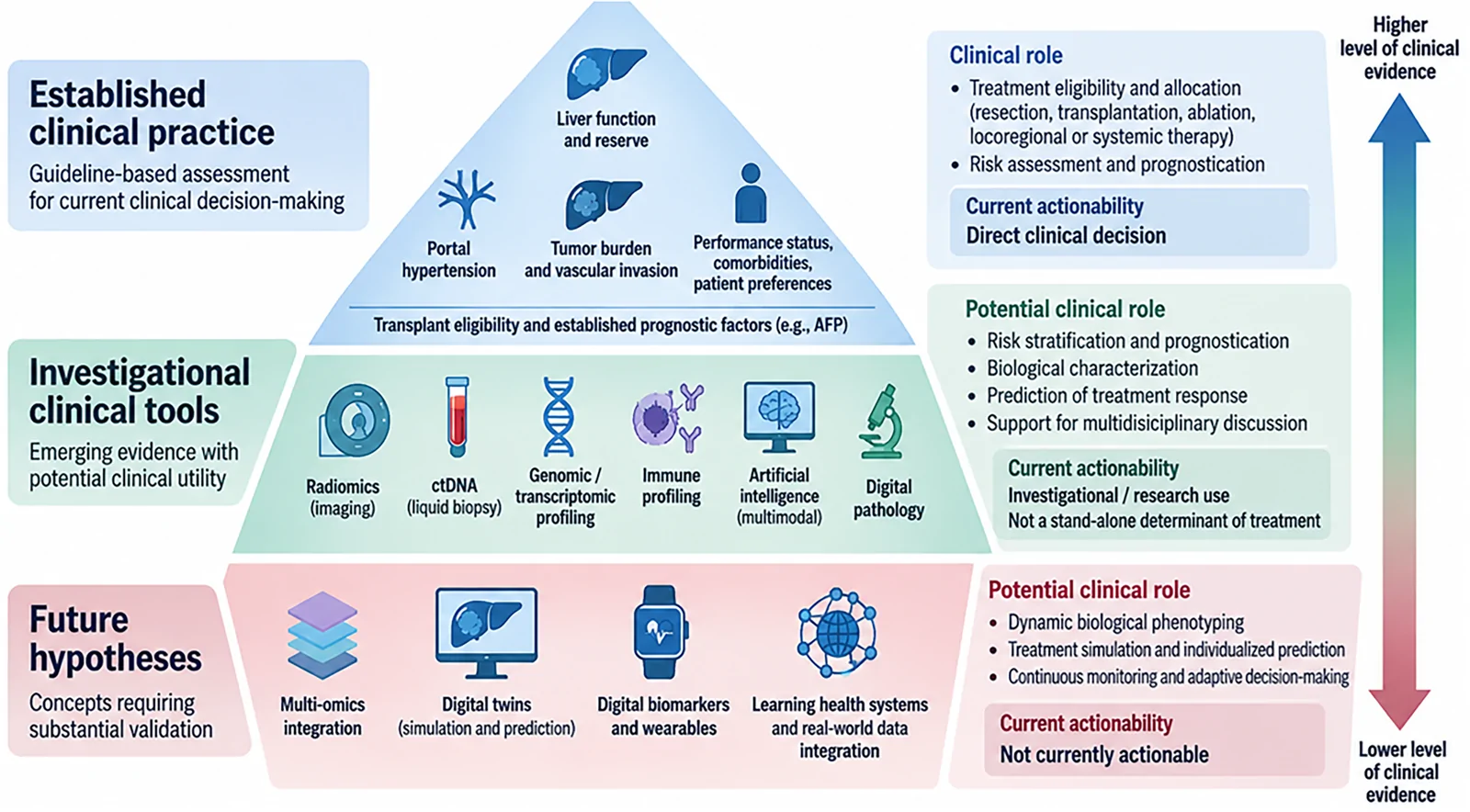
Evidence tiers of precision technologies in hepatocellular carcinoma. Precision technologies are classified into three evidence tiers according to their current level of clinical validation and actionability. Established clinical practice, including assessment of liver function, portal hypertension, tumor burden, performance status, and treatment eligibility, forms the foundation of HCC management. Investigational clinical tools, including radiomics, circulating tumor DNA (ctDNA), genomic and immune profiling, artificial intelligence (AI), and digital pathology, may provide additional information for risk stratification, biological characterization, treatment-response assessment, or multidisciplinary discussion, but are not established stand-alone determinants of treatment. Future hypotheses, including multi-omics integration, digital twins, digital biomarkers, and learning health systems, require substantial prospective validation before routine clinical implementation. Precision technologies are therefore intended to complement, rather than replace, guideline-based HCC management.

### 5.1 Radiomics

Radiomics enables quantitative extraction of imaging features that may reflect tumor heterogeneity, microvascular invasion (MVI), differentiation, recurrence risk, and treatment response (11, 13). However, its clinical readiness remains investigational. Radiomic features can be substantially influenced by image acquisition, contrast timing, segmentation, and analytical methodology, limiting reproducibility across institutions (16). Recent systematic evidence also demonstrates substantial heterogeneity among radiomics and AI models evaluated for HCC, emphasizing the need for standardized reporting and robust external validation (17). Accordingly, radiomics should currently be considered a risk-stratification tool rather than an established determinant of surgical extent or treatment selection.

### 5.2 Artificial intelligence

Artificial intelligence (AI) can integrate imaging, laboratory, pathological, and clinical variables to predict HCC detection, recurrence, postoperative liver failure, and treatment outcomes (12). Nevertheless, high retrospective predictive performance does not equal demonstrated clinical utility. Many published models remain retrospective, use selected populations, and lack prospective multicenter validation, calibration assessment, clinically meaningful comparators, or evidence that AI-guided decisions improve patient outcomes (18). AI should therefore currently function primarily as investigational decision support rather than an autonomous treatment-selection system.

### 5.3 Digital pathology and computational pathology

Whole-slide imaging and computational pathology enable quantitative assessment of tumor architecture, differentiation, vascular invasion, and molecular phenotypes. Recent studies demonstrate that AI-derived histopathological features can provide prognostic information beyond conventional pathological variables (19). However, most evidence remains retrospective or proof-of-concept, and standardized workflows, external validation, interpretability, and clinically actionable thresholds remain insufficient for routine biomarker-guided management.

### 5.4 Multimodal data integration

Integration of imaging, pathology, genomics, liquid biopsy, and clinical variables may provide a more comprehensive representation of HCC biology than any single modality. Such multimodal systems are increasingly being explored for individualized risk prediction and treatment recommendation (20). However, their feasibility should not be interpreted as evidence of clinical superiority over established staging systems. Multimodal models require prospective evaluation against guideline-based multidisciplinary decision-making and demonstration of incremental clinical benefit.

### 5.5 Current limitations of emerging technologies

Several barriers currently limit translation into routine HCC care: assay and imaging standardization, reproducibility, external and prospective validation, clinically defined decision thresholds, interoperability, cost, regulatory oversight, and equitable access. Data heterogeneity and algorithmic bias may further limit generalizability across populations and healthcare systems (17, 18). Consequently, these technologies should presently be regarded as investigational clinical tools, with their principal roles being biological characterization, risk stratification, hypothesis generation, and clinical-trial development rather than replacement of established HCC management.

## 6 Clinical translation: from guideline-based care to precision surgical oncology

The clinical translation of Precision Surgical Oncology should proceed from, rather than replace, established guideline-based HCC management. To avoid conflating biological prediction with clinical actionability, emerging approaches can be considered according to three evidence tiers: established clinical practice, investigational clinical tools, and future hypotheses.

### 6.1 Tier 1: established clinical practice

#### 6.1.1 Guideline-based patient assessment

Established assessment begins with liver function, portal hypertension, tumor burden, performance status, and technical resectability. These parameters determine whether hepatic resection, transplantation, ablation, locoregional therapy, or systemic treatment is appropriate (2–4, 10).

#### 6.1.2 Established treatment selection

Treatment selection remains primarily guideline-based and depends on the interaction between tumor stage, liver reserve, transplant eligibility, and patient characteristics (2–4). Precision information should therefore complement, rather than substitute for, these established criteria.

#### 6.1.3 Patient-centered and multidisciplinary decision-making

A multidisciplinary team should integrate disease characteristics, expected benefits and risks, comorbidities, functional status, and patient preferences. Current guidelines emphasize individualized multiparametric assessment rather than reliance on a single biomarker or technology (2, 3).

### 6.2 Tier 2: investigational clinical tools

For each emerging tool, four elements should be distinguished: clinical decision, target population, validation status, and actionability.

#### 6.2.1 Radiomics

Clinical decision: recurrence or MVI risk stratification.

Population: selected patients undergoing resection or ablation.

Validation: predominantly retrospective, although some multicenter external-validation studies are emerging (16, 17).

Actionability: currently limited. Radiomics may refine risk assessment but should not independently determine surgical extent or treatment selection.

#### 6.2.2 ctDNA and liquid biopsy

Clinical decision: MRD and recurrence-risk assessment.

Population: postoperative or biologically high-risk patients.

Validation: investigational, with substantial variation in assay sensitivity and methodology (15).

Actionability: currently insufficient to mandate treatment modification; its principal roles are research, clinical-trial enrollment, and potentially supportive information during MDT discussion.

#### 6.2.3 Molecular and immune profiling

Clinical decision: biological characterization and potential treatment-response stratification.

Population: selected patients undergoing molecular or immune profiling.

Validation: incomplete (11, 21).

Actionability: predominantly investigational and potentially useful for clinical-trial selection rather than routine surgical decision-making.

#### 6.2.4 AI and predictive modeling

Clinical decision: risk prediction, recurrence estimation, and treatment-response assessment.

Population: selected HCC cohorts with available multimodal data.

Validation: predominantly retrospective, with limited prospective clinical evaluation (18).

Actionability: decision support only; AI should not autonomously determine treatment selection.

#### 6.2.5 Digital pathology

Clinical decision: quantitative assessment of differentiation, molecular phenotype, and vascular invasion.

Population: patients with digitized pathological specimens.

Validation: early external validation is emerging, but prospective clinical utility remains unproven (19, 20).

Actionability: currently limited to prognostic or investigational applications.

Thus, these tools should presently be regarded as clinical adjuncts for risk stratification and biological characterization, not replacements for established HCC treatment pathways (22).

## 7 Precision surgical oncology across the HCC treatment continuum

Precision Surgical Oncology should be applied within, rather than outside, established HCC treatment pathways. Treatment selection should first be based on guideline-informed assessment of liver function, portal hypertension, tumor burden, vascular involvement, extrahepatic disease, performance status, technical resectability, and patient preferences. Precision tools may subsequently provide additional information for biological and risk refinement, but their ability to change treatment selection or surgical strategy remains investigational. **Table 2** summarizes established treatment pathways and illustrates how emerging precision approaches may complement, rather than replace, conventional multidisciplinary decision-making.

**Table 2:** Established HCC treatment pathways and potential precision refinement.

Established treatment pathway	Primary clinical determinants	Potential precision refinement	Current evidence status
Hepatic resection	Liver function, portal hypertension, tumor burden, vascular involvement, technical resectability, performance status	Refinement of recurrence-risk assessment, MVI risk, biological aggressiveness, and perioperative risk using radiomics, biomarkers, AI, and host factors	Established treatment; precision refinement remains investigational
Liver transplantation	Liver function, tumor burden, transplant criteria, portal hypertension, extrahepatic disease, patient suitability	Biological response, AFP dynamics, and emerging molecular or circulating biomarkers may refine selection and post-treatment assessment	Established treatment pathway; most biological refinements remain investigational
Ablation	Tumor size, number, location, liver function, and technical feasibility	Imaging-derived tumor phenotype and biological risk may support patient selection and recurrence-risk stratification	Established treatment; precision refinement remains investigational
Locoregional therapy	Tumor burden, vascular involvement, liver function, extrahepatic disease, and treatment feasibility	Imaging phenotype, treatment-response assessment, and emerging biomarkers may help refine treatment selection and sequencing	Established treatment; precision refinement remains investigational
Systemic therapy	Tumor stage, liver function, performance status, prior treatment, and treatment eligibility	Molecular, immune, and circulating biomarkers may refine biological characterization and treatment-response assessment	Established treatment; biomarker-guided selection remains limited and investigational
Conversion surgery/multimodality treatment	Initial unresectability, treatment response, residual disease, liver function, and technical resectability	Dynamic imaging, biological response, ctDNA, and AI-based prediction may potentially assist assessment of treatment response and timing of reassessment for surgery	Promising but not yet standardized; precision-guided selection remains investigational
Surveillance and recurrence management	Tumor stage, recurrence risk, liver disease status, and established imaging/laboratory surveillance	Longitudinal biomarkers, radiomics, AI, and digital monitoring may refine recurrence-risk assessment and surveillance intensity	Established surveillance principles; emerging tools remain investigational

The potential role of precision technologies therefore differs across the HCC treatment continuum. In resection and transplantation, for example, biological and molecular information may eventually refine recurrence-risk assessment or treatment sequencing, whereas current candidacy remains primarily determined by established clinical criteria. Similarly, radiomics, ctDNA, molecular profiling, and AI may support risk stratification or multidisciplinary discussion, but should not presently be interpreted as stand-alone determinants of operative extent or treatment indication. Conversion surgery and biomarker-guided perioperative strategies remain areas of active investigation requiring prospective validation.

### 7.1 Initial treatment selection

Precision Surgical Oncology should begin with established guideline-based assessment and subsequently incorporate biological and host-related information. Thus, treatment selection can be conceptualized as a sequential process of guideline assessment, biological refinement, and multidisciplinary discussion, rather than biomarker-driven replacement of conventional clinical criteria (2–4).

### 7.2 Liver resection

For patients considered for hepatic resection, biological information should supplement, but not replace, established assessment of liver functional reserve, portal hypertension, tumor burden, vascular involvement, resectability, and future liver remnant (2–4). Emerging biomarkers may refine recurrence-risk estimation; however, there is currently insufficient evidence to use predicted MVI or other biological signatures as independent determinants of wider resection or operative extent (12, 13).

### 7.3 Liver transplantation

Liver transplantation represents a particularly important setting for precision selection because both tumor recurrence and limited organ availability must be considered. Conventional selection remains centered on validated criteria, including the Milan criteria, tumor burden, liver function, and transplant eligibility (10). Downstaging with locoregional therapy can allow selected patients initially beyond standard criteria to become transplant candidates, with treatment response providing additional information regarding tumor biology (23). AFP and other biological characteristics may further refine risk stratification, but their application varies across transplant systems and should complement rather than replace established criteria (10, 23). Molecular profiling and ctDNA are promising approaches for assessing occult disease and recurrence risk, but remain investigational and are not established listing criteria.

### 7.4 Ablation and locoregional therapy

Ablation and locoregional therapies remain established components of HCC management for appropriately selected patients (2–4). Their role within Precision Surgical Oncology is primarily to provide disease control, facilitate curative-intent treatment, or contribute to downstaging and treatment sequencing.

### 7.5 Conversion surgery

Conversion surgery refers to curative-intent surgical resection performed after an initially unresectable HCC becomes technically amenable to surgery following systemic or locoregional treatment. Emerging evidence suggests that selected patients may achieve meaningful responses and undergo potentially curative resection (24). However, optimal patient selection, treatment duration, timing of surgery, and comparative benefit remain incompletely established (24, 25). Therefore, conversion surgery should be individualized through multidisciplinary assessment rather than determined by response alone.

### 7.6 Perioperative and adjuvant therapy

Perioperative and adjuvant systemic therapy remains an evolving area. Importantly, the updated IMbrave050 analysis showed that the initial recurrence-free survival benefit of adjuvant atezolizumab plus bevacizumab was not sustained (RFS HR, 0.90), while overall survival remained immature; consequently, the updated benefit-risk profile does not support routine adjuvant use (9). Precision oncology may eventually identify patients who benefit from perioperative therapy, but biomarker-guided perioperative treatment is not currently established clinical practice.

### 7.7 Postoperative surveillance and recurrence management

Postoperative surveillance should remain based on established imaging and clinical follow-up strategies (2–4). ctDNA and other liquid-biopsy approaches may provide additional recurrence-risk information, but remain investigational (15). Recurrence should be reassessed according to tumor extent, liver function, prior treatment, and patient condition, with repeat resection, ablation, locoregional therapy, transplantation, or systemic therapy considered when appropriate. Thus, precision information should support longitudinal risk assessment without replacing established recurrence-management pathways.

## 8 The proposed precision surgical oncology framework

The proposed Precision Surgical Oncology framework follows a sequential and iterative process in which guideline-based clinical assessment forms the foundation, followed by biological and host-risk refinement, multidisciplinary interpretation, individualized treatment within established pathways, and longitudinal reassessment (**Figure 3**). Emerging precision technologies are therefore used as adjunctive information rather than stand-alone determinants of treatment.

**Figure 3:**
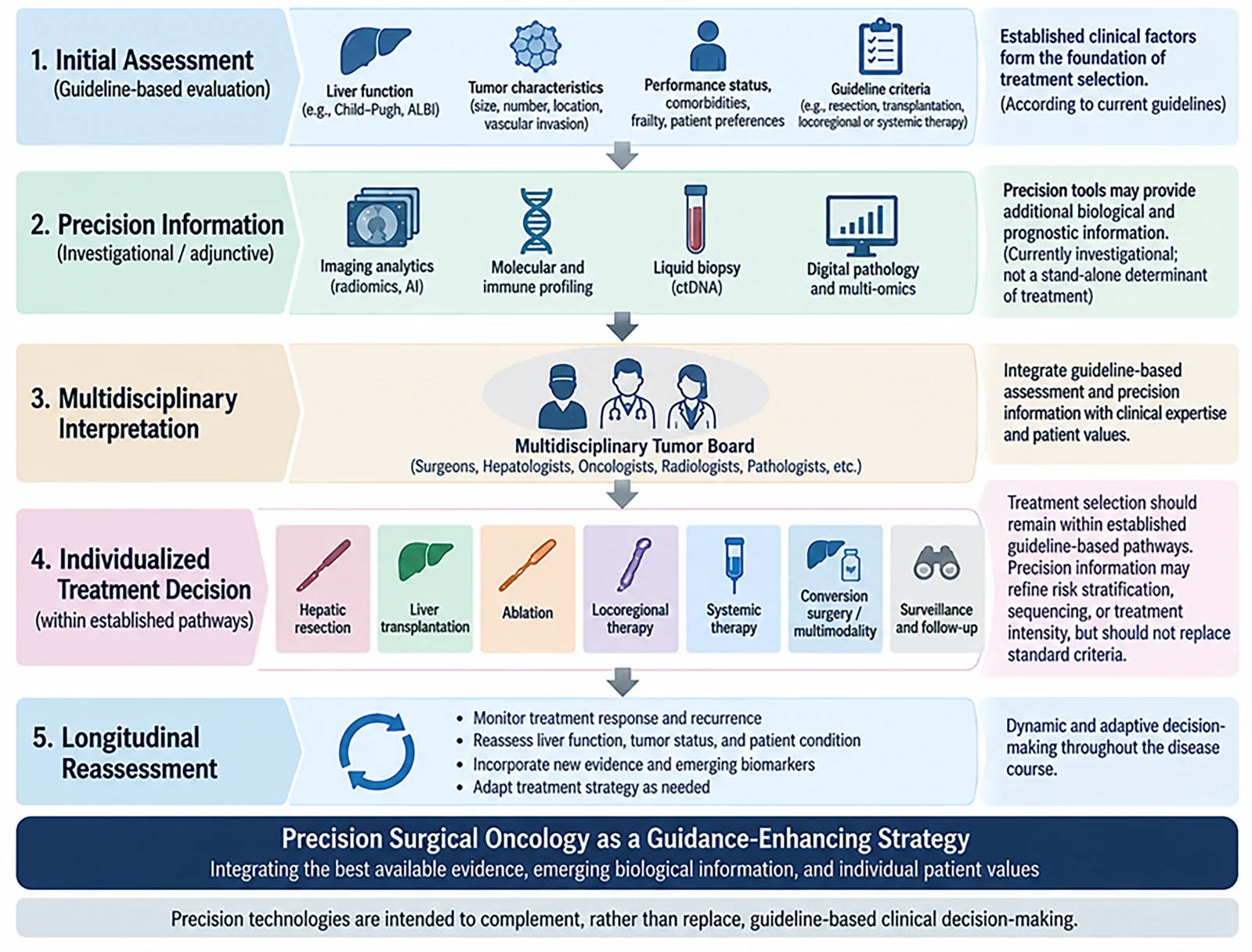
Guideline-based clinical decision algorithm for precision surgical oncology in hepatocellular carcinoma. The proposed framework consists of five sequential and iterative steps. First, guideline-based clinical assessment evaluates liver function and reserve, tumor characteristics, portal hypertension, performance status, comorbidities, frailty, and patient preferences to establish treatment eligibility. Second, emerging precision technologies may provide additional information on tumor biology and risk, including imaging analytics, molecular and immune profiling, liquid biopsy, and digital pathology. Third, these data are interpreted within a multidisciplinary team rather than used as stand-alone determinants of treatment. Fourth, individualized treatment is selected within established therapeutic pathways, including hepatic resection, liver transplantation, ablation, locoregional therapy, systemic therapy, conversion or multimodality treatment, and surveillance. Finally, longitudinal reassessment incorporates treatment response, recurrence, liver function, and evolving patient status to support adaptive clinical decision-making. The framework emphasizes that Precision Surgical Oncology complements, rather than replaces, guideline-based HCC management.

### 8.1 Principle: precision supplements rather than replaces guidelines

The central principle of Precision Surgical Oncology is that precision information should supplement, rather than replace, established guideline-based HCC management. Contemporary HCC frameworks increasingly emphasize individualized, multidisciplinary decision-making that incorporates tumor burden, liver function, biological characteristics, patient vulnerability, and treatment goals while maintaining an evidence-based foundation (2–4, 26). The 2026 BCLC update similarly emphasizes a flexible, human-centered approach in which uncertainty, biological and psychosocial factors, and patient preferences are incorporated into iterative multidisciplinary decisions (26). Recent consensus work such as BEACON-HCC further illustrates the movement toward biologically informed treatment allocation, although its recommendations remain consensus-based and require further empirical validation (27).

### 8.2 Step 1: guideline-based clinical assessment

The process begins with established clinical assessment, including liver function and reserve, portal hypertension, tumor number and size, vascular involvement, extrahepatic disease, performance status, frailty, and technical resectability. These parameters determine the initial evidence-based treatment pathway, including hepatic resection, transplantation, ablation, locoregional therapy, systemic therapy, or appropriate combinations (2–4).

### 8.3 Step 2: biological and host risk refinement

Emerging information may then refine, but not override, the initial assessment. Radiomics, ctDNA, molecular and immune profiling, AI-based prediction, and digital pathology may provide additional information regarding recurrence risk, tumor aggressiveness, treatment response, or host vulnerability (12–15, 20–22). However, these tools remain heterogeneous in validation and actionability. Their current role should therefore be regarded primarily as risk refinement, multidisciplinary discussion, or trial-enrollment support, rather than as independent determinants of surgical extent or treatment selection.

### 8.4 Step 3: multidisciplinary interpretation

Precision information should be interpreted within a multidisciplinary team rather than by an isolated algorithm or biomarker. Surgeons, hepatologists, medical and radiation oncologists, radiologists, pathologists, and other specialists should evaluate the strength, uncertainty, and applicability of each information source. This approach is consistent with contemporary HCC decision frameworks emphasizing structured, evidence-based, and patient-centered multidisciplinary deliberation (2, 26, 27).

### 8.5 Step 4: individualized treatment selection

Treatment is subsequently selected according to the integrated assessment of guideline-defined disease status, liver reserve, tumor biology, treatment feasibility, expected benefit, and patient goals. Importantly, an investigational biomarker should influence management only when its intended clinical action, target population, validation status, and actionability are sufficiently established. Otherwise, it should remain supportive or hypothesis-generating information.

Recent studies in liver malignancies further illustrate how functional drug-response assessment and tumor–immune interactions may contribute to individualized treatment strategies. In hepatocellular carcinoma (HCC), patient-derived organotypic tumor spheroids (PDOTS) have been developed as an immune-competent ex vivo platform for evaluating responses to immunotherapy and potentially guiding individualized treatment selection (28). In addition, preclinical studies have suggested that tyrosine kinase inhibition may enhance PD-1 blockade in HCC by remodeling the tumor immune microenvironment and promoting CD8+ T-cell infiltration (29). In intrahepatic cholangiocarcinoma, PDOT and patient-derived xenograft models have further implicated AKT-associated signaling in therapeutic resistance and demonstrated a potential strategy for enhancing gemcitabine sensitivity (30). Collectively, these findings support the broader concept that future precision oncology may incorporate functional drug sensitivity and dynamic tumor–immune interactions into treatment selection. However, differences in tumor type, treatment regimen, experimental model, and level of clinical validation should be recognized, and these approaches remain investigational.

### 8.6 Step 5: longitudinal reassessment

Precision Surgical Oncology is therefore conceived as a dynamic rather than one-time classification system. Tumor response, recurrence, liver function, treatment tolerance, frailty, patient preferences, and newly available biological information should be reassessed over time. Treatment decisions can consequently be modified as the clinical state evolves, consistent with the iterative decision-making principles emphasized in contemporary HCC frameworks (26).

### 8.7 Evidence tiers within the framework

To prevent premature clinical implementation, emerging technologies are classified into three evidence tiers. Established clinical practice includes guideline-supported assessment and treatment pathways with demonstrated clinical utility. Investigational clinical tools include radiomics, ctDNA, molecular/immune profiling, AI, and digital pathology; for each, the intended clinical decision, target population, validation status, and actionability should be explicitly defined before clinical implementation (20–22). Future hypotheses include multimodal data integration, digital biomarkers, AI-enabled adaptive systems, and other learning-health-system approaches whose clinical utility remains to be established. Thus, the proposed Precision Surgical Oncology framework is an author-developed, hypothesis-generating model designed to organize emerging information around established HCC care rather than to substitute for current clinical guidelines.

## 9 Future vision: toward an adaptive learning surgical oncology system

The future of Precision Surgical Oncology may extend beyond the integration of currently available biomarkers and predictive models toward a continuously learning, multimodal system. These concepts should be regarded as future hypotheses rather than established clinical capabilities.

### 9.1 Digital twins

Digital twins may eventually provide dynamic virtual representations of individual patients by integrating longitudinal clinical, imaging, pathological, molecular, and treatment-response data. Such systems could theoretically simulate alternative treatment strategies, predict changing disease trajectories, and support scenario-based decision-making (31). However, clinical validation, data integration, computational scalability, and regulatory governance remain major barriers.

### 9.2 Multimodal AI

Future AI systems may integrate imaging, pathology, clinical variables, and molecular information to generate more comprehensive representations of HCC biology than single-modality models (32). Although multimodal approaches have demonstrated promising predictive performance, their clinical utility, generalizability, interpretability, and robustness to data drift require prospective evaluation before routine clinical deployment.

### 9.3 Multi-omics integration

Integration of genomic, transcriptomic, proteomic, epigenomic, and spatial information may enable increasingly detailed characterization of tumor heterogeneity and treatment resistance. Combined with AI, multi-omics could eventually support dynamic biological phenotyping and individualized treatment simulation (32). At present, however, the complexity, cost, standardization, and external validation requirements of such approaches limit their clinical applicability.

### 9.4 Digital biomarkers and wearables

Future digital biomarkers derived from wearable devices, continuous physiological monitoring, patient-reported outcomes, and behavioral data may provide longitudinal information on functional status, treatment tolerance, recovery, and host resilience. Their potential value lies not in replacing conventional clinical assessment but in continuously capturing patient states between clinical encounters.

### 9.5 Learning health systems

A future Precision Surgical Oncology system could connect clinical practice, multimodal data, evidence generation, and guideline refinement in a continuous learning cycle. Such Learning Health Systems could progressively identify practice variation, generate hypotheses, evaluate outcomes, and inform subsequent decision-making (33, 34). Importantly, this concept requires robust governance, interoperability, data quality, and clinician engagement.

### 9.6 Real-world data and continuous evidence generation

Longitudinal real-world data may eventually complement randomized trials by capturing heterogeneous patients, treatment sequences, and outcomes encountered in routine practice. Continuous evidence-generation frameworks could create feedback loops linking real-world data to research priorities, guideline evaluation, and shared decision-making (35). Thus, the ultimate vision is an adaptive learning surgical oncology system in which evidence, clinical experience, patient-generated data, and emerging biological information continuously inform one another. This remains a future hypothesis and should not be interpreted as current clinical guidance.

## 10 Challenges, limitations, and ethical considerations

Despite its potential, Precision Surgical Oncology faces substantial biological, technical, clinical, economic, and ethical challenges. These limitations must be addressed before emerging technologies can be incorporated into routine decision-making.

### 10.1 Biological and technical limitations

HCC is biologically heterogeneous, and currently available biomarkers incompletely capture tumor evolution, host biology, and treatment resistance (11–15). Radiomics, AI, and multimodal models are also sensitive to differences in imaging acquisition, segmentation, annotation, and data quality (16, 20). Consequently, apparently high predictive performance may not translate across institutions or populations.

### 10.2 Lack of prospective clinical validation

Most emerging precision tools remain supported predominantly by retrospective or selected cohorts (16–22). External validation, calibration, prospective multicenter evaluation, and demonstration of clinical utility are essential before such tools can influence treatment selection. Importantly, prognostic performance alone does not establish that biomarker-guided management improves patient outcomes.

### 10.3 Cost and health inequity

Advanced molecular profiling, digital pathology, high-performance computing, and longitudinal data infrastructure may increase healthcare costs and may be disproportionately available in well-resourced institutions. Unequal access could therefore widen existing disparities in cancer care. Precision oncology should consequently be evaluated not only by predictive accuracy but also by affordability, accessibility, and population-level benefit.

### 10.4 Data interoperability and standardization

Integration of imaging, pathology, genomics, clinical records, and patient-generated data requires standardized definitions, acquisition protocols, data formats, and interoperable infrastructure (31–35). Without harmonization, models may learn institution-specific patterns rather than reproducible biological signals. Continuous updating also creates additional challenges related to data drift and model recalibration.

### 10.5 Regulatory and cybersecurity issues

AI systems used for clinical decision support or surgical planning raise questions regarding regulatory classification, responsibility for errors, post-deployment monitoring, and adaptive model modification (36). Connected devices and digital platforms additionally create cybersecurity and privacy risks, including unauthorized access, data manipulation, and attacks that could compromise clinical safety (37).

### 10.6 Algorithmic bias and explainability

AI models may reproduce or amplify disparities when training data underrepresent particular populations (38). Bias can arise from data collection, labeling, model development, deployment, or differences between development and clinical populations. Explainability and transparent reporting are therefore important, particularly when AI outputs influence high-stakes surgical decisions (36, 39).

### 10.7 Ethical issues in AI-guided surgical decision-making

AI should augment rather than replace multidisciplinary clinical judgment. Patient autonomy, informed consent, accountability, privacy, transparency, and the right to understand how recommendations are generated must remain central to care (36, 39). Ultimately, Precision Surgical Oncology should be implemented as a clinician-supervised, patient-centered framework, in which technological sophistication is subordinate to clinical evidence, patient values, safety, and equity.

## 11 Conclusions

Precision Surgical Oncology does not replace guideline-based HCC management. Rather, it provides a conceptual framework for integrating emerging biological and digital information with established clinical assessment and multidisciplinary decision-making.

The proposed approach follows a continuous sequence: guideline-based assessment → biological refinement → multidisciplinary interpretation → individualized treatment → longitudinal reassessment. Established clinical factors, including liver reserve, portal hypertension, tumor burden, resectability, performance status, and patient preferences, should remain the foundation of treatment selection. Emerging biomarkers and digital technologies may progressively refine risk assessment and clinical interpretation as their validation and actionability improve.

This framework therefore emphasizes not the replacement of conventional HCC treatment algorithms, but their evolution toward more individualized, adaptive, and patient-centered care. Future advances in multimodal AI, multi-omics, digital biomarkers, and learning health systems may further expand this approach, but these technologies should remain subject to rigorous prospective validation.

Ultimately, Precision Surgical Oncology is an author-proposed, hypothesis-generating framework intended to complement, rather than substitute for, evidence-based HCC management.
